# Expanding the CRISPR Toolbox in *P. patens* Using SpCas9-NG Variant and Application for Gene and Base Editing in *Solanaceae* Crops

**DOI:** 10.3390/ijms21031024

**Published:** 2020-02-04

**Authors:** Florian Veillet, Laura Perrot, Anouchka Guyon-Debast, Marie-Paule Kermarrec, Laura Chauvin, Jean-Eric Chauvin, Jean-Luc Gallois, Marianne Mazier, Fabien Nogué

**Affiliations:** 1 INRAE, Agrocampus Ouest, Université de Rennes, IGEPP, F-29260 Ploudaniel, France; marie-paule.kermarrec@inrae.fr (M.-P.K.); laura.chauvin@inrae.fr (L.C.); jean-eric.chauvin@inrae.fr (J.-E.C.); 2 Germicopa Breeding, Kerguivarch, 29520 Chateauneuf Du Faou, France; 3 INRAE, GAFL, F-84143 Montfavet, France; laura.perrot@inrae.fr (L.P.); jean-luc.gallois@inrae.fr (J.-L.G.); marianne.mazier@inrae.fr (M.M.); 4 Institut Jean-Pierre Bourgin, INRAE, AgroParisTech, Université Paris-Saclay, 78000 Versailles, France; anouchka.guyon@inrae.fr

**Keywords:** CRISPR-Cas9, base editing, CBE, alternative PAM, xCas9, SpCas9-NG, *Physcomitrella patens*, potato, tomato

## Abstract

Genome editing has become a major tool for both functional studies and plant breeding in several species. Besides generating knockouts through the classical CRISPR-Cas9 system, recent development of CRISPR base editing holds great and exciting opportunities for the production of gain-of-function mutants. The PAM requirement is a strong limitation for CRISPR technologies such as base editing, because the base substitution mainly occurs in a small edition window. As precise single amino-acid substitution can be responsible for functions associated to some domains or agronomic traits, development of Cas9 variants with relaxed PAM recognition is of upmost importance for gene function analysis and plant breeding. Recently, the SpCas9-NG variant that recognizes the NGN PAM has been successfully tested in plants, mainly in monocotyledon species. In this work, we studied the efficiency of SpCas9-NG in the model moss *Physcomitrella* *patens* and two *Solanaceae* crops (*Solanum lycopersicum* and *Solanum tuberosum*) for both classical CRISPR-generated gene knock-out and cytosine base editing. We showed that the SpCas9-NG greatly expands the scope of genome editing by allowing the targeting of non-canonical NGT and NGA PAMs. The CRISPR toolbox developed in our study opens up new gene function analysis and plant breeding perspectives for model and crop plants.

## 1. Introduction

Genome editing via the versatile and efficient CRISPR-Cas9 system has emerged as a major tool for both functional studies and plant breeding in several species [1]. To fulfil its function, this two-component system relies on a customized single-guide RNA (sgRNA) that, by recruiting the Cas9 endonuclease, drives the targeted double strand DNA break (DSB). The specificity of the CRISPR-Cas9 depends on both the complementarity between the sgRNA and the target DNA, and the protospacer adjacent motif (PAM) that is recognized by the Cas9 and allows for subsequent DNA:RNA pairing. For the *Streptococcus pyogenes* Cas9 (SpCas9), NGG is the canonical PAM motif [2]. More recently, base editing technologies have been developed to allow precise and predictable targeted nucleotide conversion without the introduction of a DSB. CRISPR-base editors are efficient tools for both the production of gain-of-function mutants and plant breeding.

Although the PAM requirement may not be an important issue for most gene knockout purposes because of the relative high occurrence of the NGG motif in most of plant genomes, it is a strong limitation for CRISPR technologies such as base editing, because the base conversion generally occurs in a narrow edition window at the 5′ extremity of the guide. As some agronomic traits can be conferred by a precise single amino-acid substitution, such as in *eIF4E* genes for virus resistance [3], development of Cas9 variants with relaxed PAM recognition is of upmost importance for plant breeding. Diversity in PAM requirement has been achieved by isolating some Cas9 variants among the bacterial species diversity or through protein engineering, but the resulting PAM is often more complex and/or these variants suffer from low efficiency compared with the SpCas9 in plants [1]. Recently, two SpCas9 variants, xCas9 3.7 and SpCas9-NG, have been engineered to recognize the non-canonical NGN PAM in animals [4,5] and subsequently successfully tested in the monocotyledon rice and in the dicotyledon *Arabidopsis thaliana* and tomato [6,7,8,9,10,11]. Demonstration that these SpCas9 variants can be used in Bryophytes and other dicotyledonous crops would significantly increase the potential of CRISPR-based gene editing strategies for precision breeding. 

Here, we compared the relative efficiency of SpCas9, xCas9 and SpCas9-NG in different PAM contexts using *Physcomitrella patens* as a model and showed that the SpCas9-NG targets more efficiently alternative NGT PAMs. We then studied the efficiency of SpCas9-NG in tomato (*Solanum lycopersicum*) and potato (*Solanum tuberosum*) for both classical CRISPR-generated gene knock-out and cytosine base editing, demonstrating the usefulness of this variant for genome editing in Bryophytes and *Solanaceous* crops.

## 2. Results and Discussion

### 2.1. SpCas9-NG Recognizes Non-Canonical PAMs in P. patens

**Figure 1:**
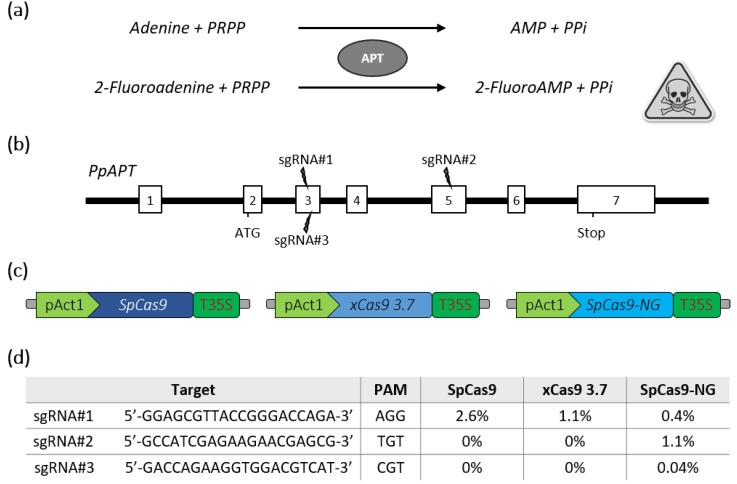
Schematic description of the *PpAPT* edition strategy and editing efficiencies for SpCas9 variants. (**a**) Adenine phosphoribosyltransferase (APT) catalyses a phosphoribosyl transfer from Phosphoribosyl Pyrophosphate (PRPP) to adenine, forming AMP and releasing pyrophosphate (PPi). In the presence of 2-Fluoroadenine APT will form 2-FluoroAMP, a toxic compound for the cell. (**b**) Structure of the *PpAPT* gene with the target sites (white rectangles represent exons). (**c**) The three constructs used for PEG-mediated Cas9 expression in *P. patens* are schematically depicted. (**d**) Results for efficiency of indel mutations using SpCas9, xCas9 3.7 and SpCas9-NG are indicated as the percentage of 2-fluoroadenine resistant protoplasts.

The moss *Physcomitrella patens* has been used as a model plant for almost 20 years. The predominant role played by this plant model in gene function analysis has been facilitated by the publication of its genome sequence [12] and the availability of various tools for the functional analysis of genes, such as the inactivation of genes obtained through gene targeting, thanks to high homologous recombination (HR) levels in this moss [13] or gene silencing mediated by RNA interference (RNAi) [14]. In recent years, the CRISPR-Cas9 [15,16,17,18] or CRISPR-Cas12a [19] systems were successfully applied to *P. patens* for targeted mutagenesis of single or multiple genes, expanding the gene analysis toolbox for this species. In order to increase the number of targets that could be modified in this model organism, we estimated the potential of the new generation of SpCas9 variants with alternative PAM sequences for gene editing. For this purpose, the *Arabidopsis* codon-optimized SpCas9 [20] was modified by DNA synthesis to produce either the xCas9 3.7 [4] or the SpCas9-NG [5]. We then targeted the reporter *PpAPT* gene in *P. patens*. Mutations leading to a loss of APT activity confer resistance to the toxic adenine analogues 2-fluoroadenine (2-FA) (Figure 1a), and thus mutation efficiency is directly proportional to the percentage of 2-FA resistant protoplasts [21]. Three guides with different PAM contexts were designed in the *PpAPT* gene (Figure 1b). The nucleases were then assayed in *P. patens* wild-type protoplasts by PEG-mediated co-transfection with two plasmids [16]: one bearing the different nucleases expressed genes (Figure 1c), and the other bearing guides targeting the *PpAPT* gene (Figure 1d). Our results indicate that the classical SpCas9 and the xCas9 can efficiently edit DNA using a canonical NGG PAM (Figure 1d). In contrast, the SpCas9-NG, while being less efficient for the editing at NGG PAM, was the more suitable variant to target the non-canonical TGT and CGT PAMs, although with rather limited efficiency for the CGT PAM (Figure 1d).

### 2.2. Targeting Indels Generation through SpCas9-NG in Potato and Tomato

**Figure 2:**
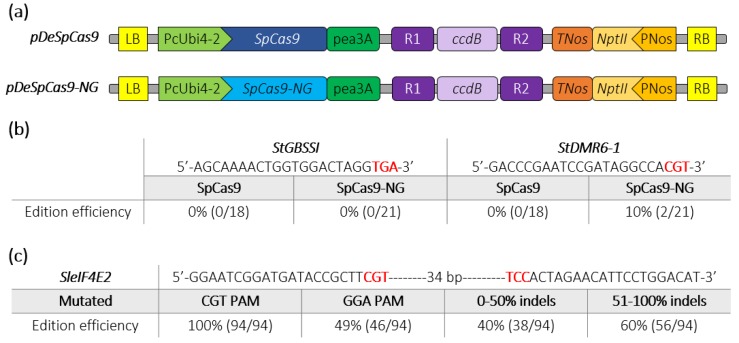
Schematic representation of SpCas9 and SpCas9-NG binary plasmids and editing efficiencies in potato and tomato. (**a**) The construct backbones used for *Agrobacterium*-mediated transformation of potato (Desiree) and tomato (WVA106) are schematically represented. The R1_ccdB_R2 cassette allows the insertion of the guide cassette through a Gateway LR reaction. (**b**) Edition efficiency represents the percentage of mutated potato plants among the transgenic plants. The PAM sequence is depicted in bold red. (**c**) Among the mutated tomato plants screened by HRM, 94 plants were Sanger sequenced and analyzed using ICE analysis. Edition efficiency represents the percentage of mutated plants with the SpCas9-NG at the *SleIF4E2* locus for each target (CGT or GGA PAMs). The mutated plants are classified into two groups according to the rate of indels at the targeted *eIF4E2* locus. The PAM sequence is depicted in bold red.

We next assayed the activity of SpCas9-NG in two crops belonging to the *Solanaceae* family. We replaced the SpCas9 by the SpCas9-NG sequence into the pDeCas9 [20], resulting in the pDeSpCas9-NG (Figure 2a). We simultaneously targeted the *StDMR6-1* (*PGSC0003DMG400000582*) and *StGBSSI* (*PGSC0003DMG400012111*) genes, which are candidate genes related to agronomic traits [22,23,24], in the tetraploid potato (*Solanum tuberosum*) cultivar “Desiree” through *Agrobacterium*-mediated transformation [23]. The target sequences for *StDMR6-1* harboured a CGT PAM while the one targeting *StGBSSI* harboured a TGA PAM not tested in *P. patens* (Figure 2b). High Resolution Melting (HRM) analysis and Sanger sequencing showed that, as expected, the SpCas9 could not induce indels at either targeted loci. In contrast, although we could not detect mutations at the sequence with the TGA PAM using the SpCas9-NG, we found that this variant was able to induce indels mutations at the target sequence harboring the CGT PAM with 10% efficiency (Figure 2b and Figure S1). Using the pDeSpCas9-NG, we then simultaneously targeted two closely located sequences with non-canonical CGT and GGA PAMs in the tomato (*Solanum lycopersicum*) *SleIF4E2* gene (*Solyc02g021550*) through *Agrobacterium*-mediated transformation. Using HRM analysis, we found that 56% of the transformed lines displayed mutations in the target sequence (Figure 2c), confirming previous results showing that the SpCas9-NG is functional in tomato [11]. Among these T0 mutated plants, genomic sequences of 94 plants were further analyzed using the ICE software (V2.0, Menlo Park, CA, USA) (https://ice.synthego.com), which determines rate, position and nature of CRISPR-Cas9 editing using Sanger chromatograms. We found that 100% of the analyzed plants were mutated at the CGT PAM targeted locus while 49% harboured mutations at the GGA PAM targeted locus albeit with a global lower rate (Figure 2c). Furthermore, mutation efficiency at the *SleIF4E2* gene was high as 60% of the plants displayed an indels score >50% at the target locus (Figure 2c). Many plants were mosaic with a variety of mutations, including indels and large deletions between the two guide sequences, while a few plants displayed a heterozygous profile, according to the ICE software (Figure S2). Taken together, these data indicate that the SpCas9-NG variant can efficiently edit target sequences with non-canonical NGT and NGA PAMs in the model plant *P. patens* and in the two crops potato and tomato.

### 2.3. Efficient Cytosine Base Editing at Non Canonical PAM in Potato and Tomato

**Figure 3:**
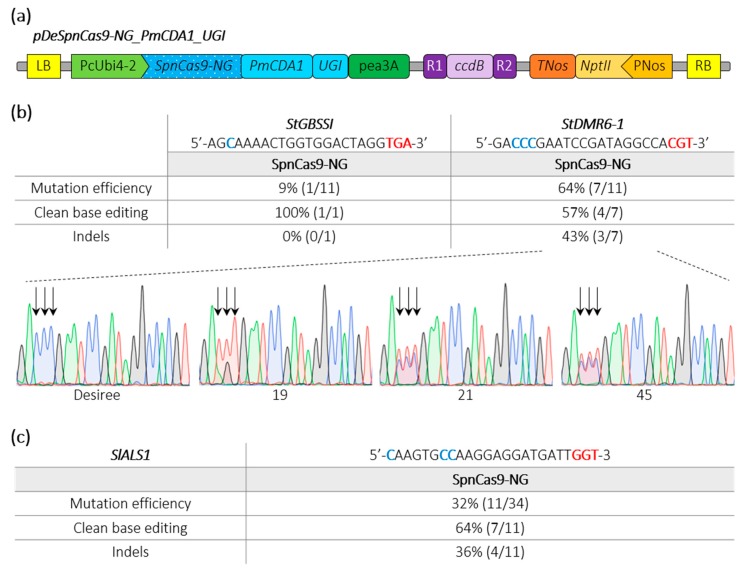
Schematic representation of a SpnCas9-NG cytosine base editing binary plasmid and editing efficiencies in potato and tomato. (**a**) The cytosine base editor construct used for *Agrobacterium*-mediated transformation of potato (Desiree) and tomato (WVA106) is schematically represented. (**b**,**c**) Edition efficiency represents the percentage of mutated plants among the transgenic plants. The modified cytosines observed in the edited plants are indicated in bold blue. Clean base editing refers to plants harboring substitution(s) without any detectable indels on Sanger chromatograms. The *StDMR6-1* Sanger chromatograms for the control potato variety (Desiree) and three potato mutants harboring clean base editing are shown. The PAM sequence is depicted in bold red. Black arrows highlight the position of edited cytosines. A, T, C and G bases are represented in green, red, blue and black, respectively.

Besides generating gene knockouts through indels, we developed cytosine base editors (CBEs) to induce precise nucleotide substitutions. In our previous studies using a CBE with a PmCDA1 cytosine deaminase devoid of uracil glycosylase inhibitor (UGI), we noticed a substantial rate of indels [3,23,25]. To improve base editing outcomes, we fused an UGI to a SpnCas9-NG (D10A), resulting into the pDeSpnCas9-NG_PmCDA1_UGI (Figure 3a). Using this CBE construct, we first targeted in potato the same loci with TGA and CGT PAMs, as done above with the pDeSpCas9-NG. We found that 64% of the transgenic plants were mutated at the target sequence with the CGT PAM (three of the seven mutants displayed indels) while only 9% of the transgenic plants displayed base editing at the locus harboring the TGA PAM (Figure 3b). 

Next, in tomato, we targeted a locus harboring a GGT PAM in the tomato *SlALS1* gene (*Solyc03g044330*) using *Agrobacterium*-mediated transformation. HRM and Sanger sequencing revealed that 32% of the transformed tomato plants were mutated in the target sequence with 64% of clean base editing corresponding to C-to-T substitution (Figure 3c). Furthermore, we also regenerated plants from a medium-containing the ALS-inhibitor chlorsulfuron, as previously described [25,26]. We regenerated 12 plants that all displayed mutations in the target sequence as revealed by HRM analysis, indicating that the CBE efficiently recognized the GGT PAM to confer resistance to the ALS inhibitor through base substitution.

**Figure 4:**
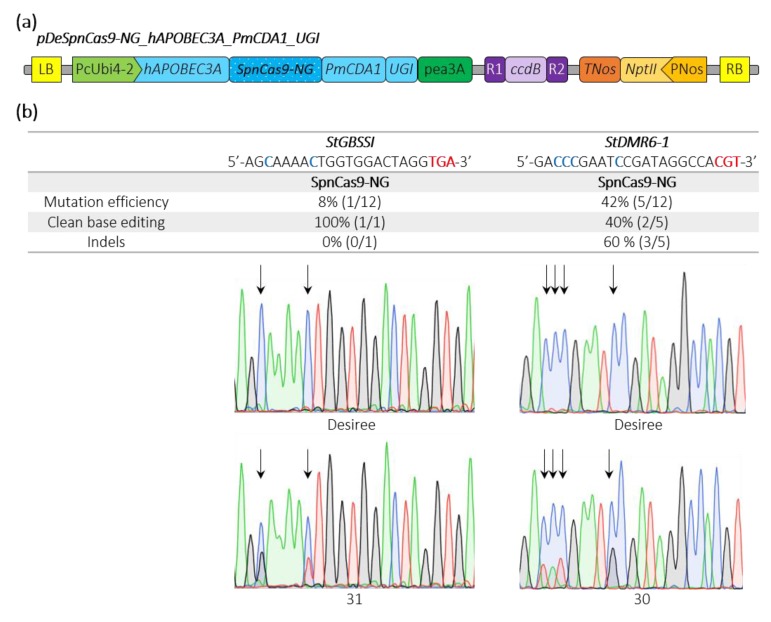
Schematic representation of a SpnCas9-NG double cytosine base editor binary plasmid and editing efficiencies in potato. (**a**) The double cytosine base editor construct used for *Agrobacterium*-mediated transformation of potato (Desiree) is schematically represented. (**b**) Edition efficiency represents the percentage of mutated plants among the transgenic plants. The modified cytosines observed in the edited plants are indicated in bold blue. Clean base editing refers to plants harboring substitution(s) without any detectable indels on Sanger chromatograms. The *StGBSSI* and *StDMR6-1* Sanger chromatograms for the control potato variety (Desiree) and two potato mutants harboring clean base editing with expanding editing window are shown. The PAM sequence is depicted in bold red. Black arrows highlight the position of edited cytosines. A, T, C and G bases are represented in green, red, blue and black, respectively.

Recently, a base editor harboring a human APOBEC3A cytosine deaminase has been shown to expand the editing window in plants [27]. We fused the hAPOBEC3A plant-codon optimized sequence to the 5′end of the SpnCas9-NG_PmCDA1_UGI, generating the pDeSpnCas9-NG_hAPOBEC3A_PmCDA1_UGI construct (Figure 4a). We simultaneously targeted the same potato loci used above and we found an editing efficiency of 42% and 8% at the sequences with the CGT and the TGA PAMs, respectively (Figure 4b). Interestingly, we found one plant harboring a C_-13_-to-T_-13_ conversion and one plant with a C_-11_-to-G_-11_ substitution at the *StGBSSI* and *StDMR6-1* targets, cytosines that were unmodified in all the edited plants using the PmCDA1 alone (Figure 3b and Figure 4b), suggesting that our double cytosine base editor construct may expand the cytosine base editing window at NGT and NGA PAMs.

## 3. Materials and Methods

### 3.1. Vector Cloning

Coding sequences of xCas9 3.7, SpCas9-NG, PmCDA1, UGI and hAPOBEC3A were plant codon-optimized and synthesized (TwistBioscience, San Francisco, CA, USA), and cloned into pAct-Cas9 [16] and pDeCas9 [28] by replacing the SpCas9 sequence. The SpnCas9-NG (D10A) sequence was obtained using PCR amplification with the Invitrogen Platinum SuperFi DNA polymerase (Thermo Fisher Scientific, Waltham, MA, USA). Sequence assembly was performed using classical restriction/ligation cloning. For *P. Patens* transfection, guide cassettes bearing the PpU6 promoter, the 20bp guide sequence and the esgRNA scaffold were synthesized (TwistBioscience, San Francisco, CA, USA). For *Agrobacterium*-mediated transformation, 20-bp guide sequences were introduced into pre-assembled entry plasmids (bearing AtU6, StU6 or SlU6 promoters, and esgRNA scaffold) using BsaI or AarI restriction sites. A Gateway LR reaction was then performed to introduce the guide cassette into the destination plasmid.

### 3.2. P. Patens PEG-Mediated Transfection

Moss protoplasts (4.8 × 10^5^) were cotransformed with the pAct-Cas9, pAct-xCas9 3.7 or pAct-SpCas9-NG and plasmids bearing guides targeting sequences with different PAM contexts in the *PpAPT* reporter gene. Non-sense mutations in the *PpAPT* gene confer resistance to the toxic adenine analogue 2-fluoroadenine (2-FA). Regenerating protoplasts were selected on PpNH4 medium supplemented with 10 µM 2-FA (Fluorochem, Hadfield, United Kingdom) to detect clones which had been disrupted at the *PpAPT* locus.

### 3.3. Agrobacterium-Mediated Transformation of Potato and Tomato Explants

The tetraploid potato cultivar Desiree (ZPC, Joure, The Netherlands) was in vitro propagated in a controlled environmental chamber at 19 °C under a 16 h light/8 h dark photoperiod and transformed as previously described [25]. Plant regeneration was performed using a selection medium containing 250 µg mL^−1^ cefotaxime (Duchefa, Haarlem, The Netherlands), 100 µg mL^−1^ timentin® (Duchefa, Haarlem, The Netherlands) and 50 µg mL^−1^ kanamycin (Duchefa, Haarlem, The Netherlands).

Plants from the WVA106 tomato cultivar were cultured in sterile conditions in a growth chamber with controlled temperatures of 22 °C/18 °C under a 16 h/8 h (day/night) photoperiod. *Agrobacterium*-mediated transformation using the C58 pGV2260 strain was performed on cotyledon segments from 8-12 day-old seedlings, as previously described [29]. Plant regeneration was performed using a selection medium containing 225 µg mL^−1^ timentin® (Duchefa, Haarlem, The Netherlands) and 100 µg mL^−1^ kanamycin (Duchefa, Haarlem, The Netherlands).

### 3.4. Genotyping Analysis

Genotyping analyses (High Resolution Melting and Sanger sequencing) for potato and tomato were performed as previously described [25].

## 4. Conclusions

Taken together, our results are in line with previous studies in plants [1], and extent to base editing recent results obtained in tomato with SpCas9-NG [11]. We showed that the SpCas9-NG greatly expands the scope of genome editing in the model plant *P. patens* and in two *Solanaceae* crops by allowing the targeting of non-canonical NGT and NGA PAMs. This possibility will increase significantly the number of amino acids that could be modified for gene function analysis purposes in *P. patens*. Indeed, when using base editing for functional analysis of the APT enzyme (manuscript in preparation), if 40% (74 over 184) of the amino acids constituting the protein can be targeted using the classical SpCas9, the totality of these amino acids can be theoretically modified using the SpCas9-NG. In the same way, use of the SpnCas9-NG with base editors in the two *Solanaceous* crops at non-canonical PAMs should allow to substantially increase the target range of base modifications. Compared to classical CBE with SpnCas9, our constructs based on the SpnCas9NG are predicted to increase more than 3 times the number of guide sequences usable for modification of *StGBSSI* and *SleIF4E2*, two important genes for quality and pathogen resistance traits [23,30], respectively. The association of base editing and higher PAM diversity open opportunities to generate more diverse alleles and possibly adjust traits of interest in these major crops. Therefore, the CRISPR toolbox developed in our study opens up new types of gene function analysis in the model moss *P. patens* and new plant breeding perspectives in *Solanaceae* crops.

## Supplementary Materials

Supplementary materials can be found at https://www.mdpi.com/1422-0067/21/3/1024/s1.

## Abbreviations

2-FA	2-fluoroadenine
ALS	Acetolactate synthase
APT	Adenine phosphoribosyltransferase
CBE	Cytosine base editor
DMR6	Downy mildew resistance 6
DSB	DNA double-strand breaks
eIF4E	Eukaryotic translation initiation factor 4E
GBSSI	Granule-bound starch synthase I
HRM	High resolution melting
PAM	Protospacer adjacent motif
UGI	Uracil DNA glycosylase inhibitor protein
