# Supplementary material for: Expanding the CRISPR Toolbox in P. patens Using SpCas9-NG Variant and Application for Gene and Base Editing in Solanaceae Crops
Source: Int J Mol Sci. 2020 Feb 4;21(3):1024. doi: 10.3390/ijms21031024 (PMC7036883; doi:10.3390/ijms21031024)
Supplement: Supplementary file 1 [file ijms-21-01024-s001.pdf]

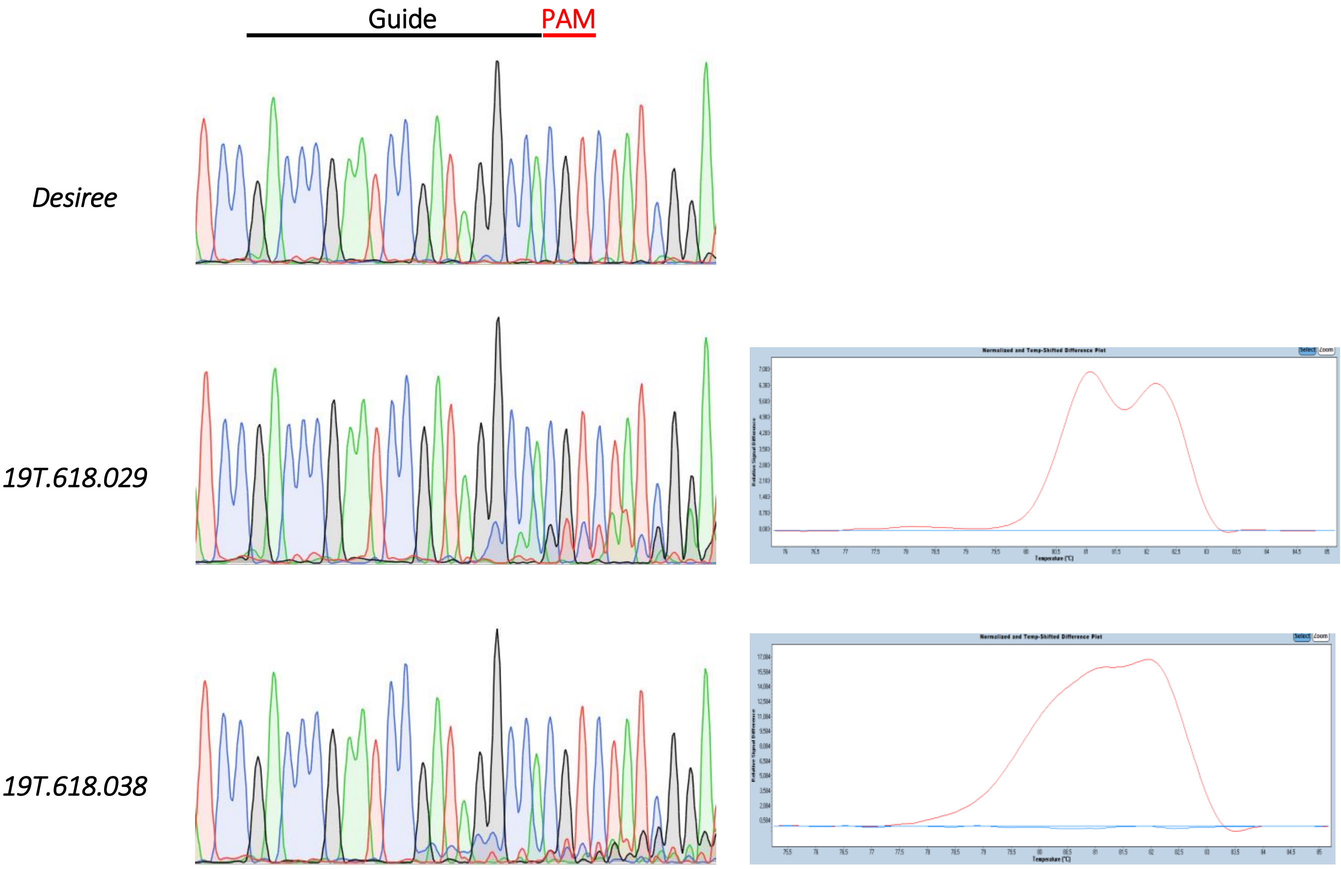

Figure S1: Sanger chromatograms and HRM analysis of the targeted *StDMR6-1* locus of the potato cultivar Desiree. For chromatograms, guide and PAM sequences are indicated in black and red, respectively. A, T, C and G bases are represented in green, red, blue and dark, respectively. For HRM analysis, Desiree samples are depicted in blue while the mutant curve is depicted in red.

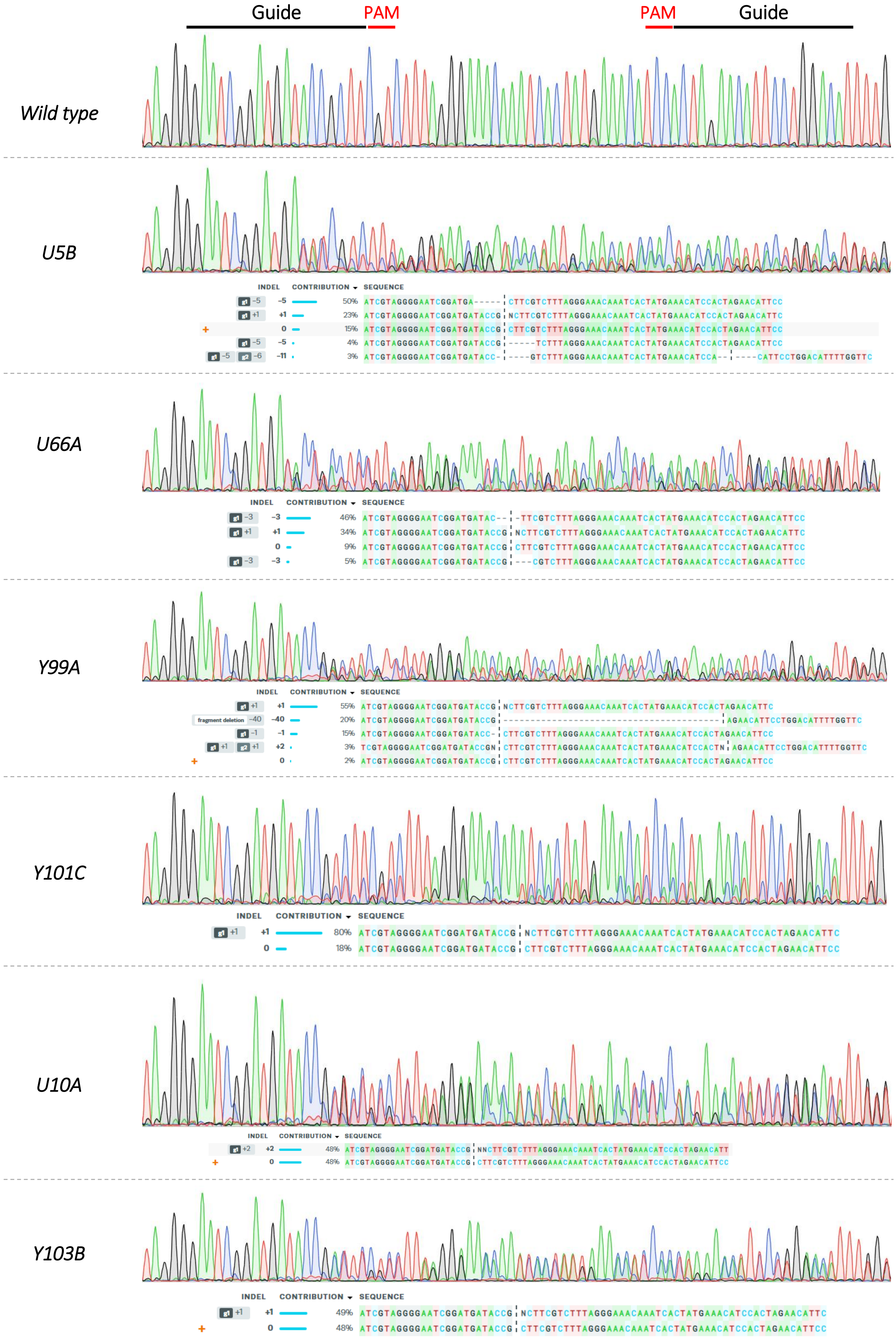

Figure S2: Sanger chromatograms and ICE analysis of the targeted *SleIF4E2* locus for some tomato plants.
For chromatograms, guide and PAM sequences are indicated in black and red, respectively. A, T, C and G bases are represented in green, red, blue and black, respectively. For ICE analysis, outputs examples are depicted with the wild-type sequence indicated by a yellow cross. Contribution for each sequence (wild-type or mutated) is represented as a percentage, with the cut site indicated on the left (g1 and/or g2).
